# Mononeuritis multiplex: an uncommon neurological manifestation of cytomegalovirus reactivation in an HIV-infected patient

**DOI:** 10.1186/s12879-018-3501-2

**Published:** 2018-11-12

**Authors:** Pedro Palma, Andreia Costa, Raquel Duro, Nélia Neves, Cândida Abreu, António Sarmento

**Affiliations:** 1 Infectious Diseases Department, Centro Hospitalar de São João, Alameda Professor Hernâni Monteiro, 4200-319 Porto, Portugal; 2 Instituto de Inovação e Investigação em Saúde (I3S). Grupo de I&D em Nefrologia e Doenças Infeciosas, Instituto Nacional de Engenharia Biomédica (INEB), Porto, Portugal; 3 Neurology Department, Centro Hospitalar de São João, Alameda Professor Hernâni Monteiro, 4200-319 Porto, Portugal

**Keywords:** Mononeuritis multiplex, CMV, HIV, AIDS

## Abstract

**Background:**

Cytomegalovirus (CMV) reactivation with neurological involvement in patients with acquired immunodeficiency syndrome (AIDS) is increasingly rare since the introduction of antiretroviral therapy (ART). Manifestations include encephalitis, myelitis, polyradiculopathy and, less commonly, mononeuritis multiplex (MNM). We report a case of disseminated CMV disease with gastrointestinal and peripheral and central nervous system involvement in a patient with AIDS, manifesting primarily as MNM.

**Case presentation:**

A 31-year old woman with AIDS presented with a clinical picture of MNM. Electromyography confirmed the clinical findings. CMV DNA was detected in cerebrospinal fluid (CSF) and blood. Gastrointestinal involvement was histologically documented. HIV RNA was also detected in CSF and brain MRI was consistent with HIV encephalopathy. A diagnosis of disseminated CMV disease (with esophagitis, colitis, encephalitis and MNM) and HIV encephalopathy was made. Treatment consisted of ganciclovir and foscarnet, followed by maintenance therapy with valganciclovir. Evolution was favorable and valganciclovir was stopped after sustained immune recovery following ART initiation.

**Conclusion:**

We discuss the diagnostic approach to CMV neurological disease, with a focus on MNM and CMV encephalitis. Combination therapy with ganciclovir and foscarnet should be considered for all forms of neurological involvement, although available data are scarce. Since there is significant overlap between CMV encephalitis and HIV encephalopathy, ART drugs with higher CSF penetration may have to be considered. ART and immune recovery are essential to improve outcomes.

## Background

Mononeuritis Multiplex (MNM) is an uncommon form of peripheral neuropathy, usually presenting with motor and sensory symptoms in an asymmetric pattern involving two or more peripheral nerves [1, 2]. In HIV-infected patients with MNM, two etiological mechanisms have been described: autoimmune, typically a limited form in patients without acquired immunodeficiency syndrome (AIDS); and cytomegalovirus (CMV) reactivation, a generalized form in patients with AIDS [2, 3]. CMV reactivation in HIV patients with advanced disease most frequently presents as retinitis, colitis, and esophagitis. Neurological involvement is less common and can manifest as encephalitis, myelitis, polyradiculopathy, and mononeuritis multiplex. In the pre-antiretroviral therapy (ART) era, where up to 40% of HIV-infected patients with advanced disease developed CMV disease [4, 5], CMV MNM was already considered uncommon [6, 7]. A more frequent type of neurological disease, CMV encephalitis, was reported in 1% of cases [6, 8]. Autopsy studies, however, recognized CMV infection of the central nervous system (CNS) in 12 to 28% of patients with AIDS [6, 9]. Similarly to other opportunistic infections, since the introduction of ART the incidence of CMV disease decreased considerably, with recently reported incidence rates of < 0.50 per 100 person-years [10, 11].

We describe a case of disseminated CMV disease in a patient with AIDS, manifesting primarily as MNM. We focus on the neurological involvement of CMV and discuss the diagnostic challenges and treatment approach of this increasingly uncommon manifestation.

## Case report

A 31-year-old white woman presented with severe burning pain with tingling sensation and asymmetric weakness of the lower limbs that, over a six-month period, gradually worsened and progressed to involve the upper limbs; she was then unable to walk or eat alone. She had been diagnosed with HIV infection five years earlier at another tertiary care hospital but refused follow-up.

On admission, she was undernourished and her neurological examination revealed: lethargy, disorientation and psychomotor slowing; asymmetrically diminished motor strength (Medical Research Council Scale) in the four limbs (grade 2/5 in right upper limb extension; grade 3/5 in bilateral lower limb extension; grade 4/5 in the remaining); symmetrical deep tendon reflexes apart from absent right brachioradialis and bilateral patellar reflexes; impaired pin-prick sensibility in the right ulnar and radial distribution. The remainder physical examination was unremarkable.

**Fig. 1:**
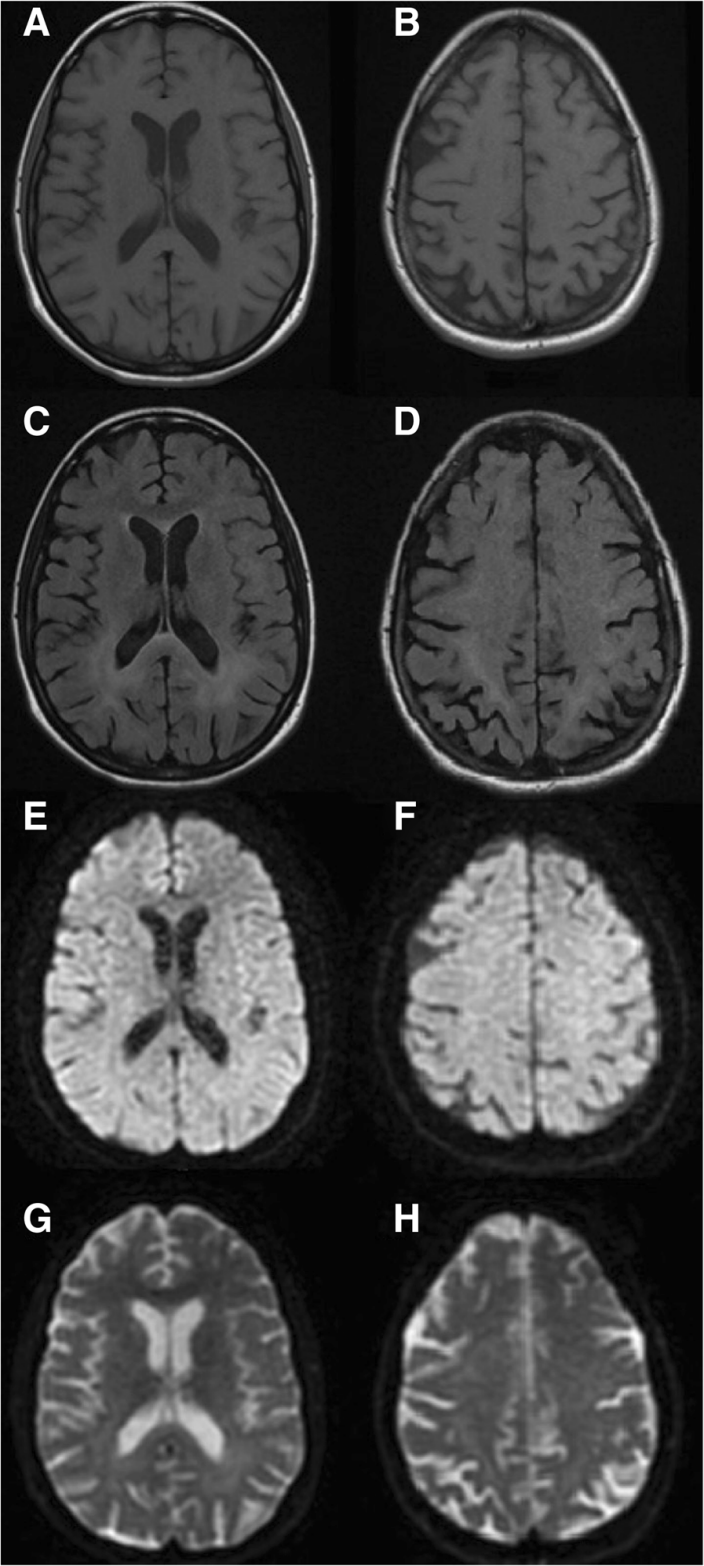
Brain MRI. Axial T1 (**a**-**b**), T2/FLAIR (**c**-**d**), DWI (**e**-**f**) and ADC-map (**g**-**h**). Bilateral relatively symmetric periventricular and deep white matter T2/FLAIR hyperintensity predominantly in the posterior supratentorial area. Areas of restricted diffusion were not observed

Brain MRI (Fig. 1) was consistent with HIV encephalopathy and electromyography (EMG) with the diagnosis of MNM (severe confluent multifocal demyelination and axonal loss in both upper and lower limbs).

CD4 cell count was 75 cells/μL (8%) and HIV RNA was 633000 copies/mL. CMV DNA in blood was 64000 copies/mL; CMV antigen was negative. CMV IgG antibodies were positive and IgM antibodies negative; furthermore, electronic medical records from five years earlier confirmed prior CMV IgG seropositivity, suggesting CMV reactivation. Cerebrospinal fluid (CSF) analysis revealed 7 cells, protein of 1.19 g/dL and glucose of 49 mg/dL; negative bacterial and fungal cultures; positive CMV DNA (14400 cp/mL) and HIV RNA (184222 cp/mL). Gastrointestinal involvement (esophageal and colonic) by disseminated CMV disease was histologically documented, even though the patient reported no related symptoms; retinal involvement was excluded.

With disseminated CMV disease as the most likely cause of MNM, she started IV ganciclovir (5 mg/kg every 12 h). Due to no significant clinical improvement, treatment was intensified 7 days later with the association of IV foscarnet (90 mg/kg every 12 h). Combination therapy was maintained for three weeks, after which CMV DNA became undetectable in blood (she refused a new lumbar puncture) and oral valganciclovir 900 mg/day was started as maintenance therapy. She initiated ART with emtricitabine/tenofovir and dolutegravir following two weeks of combination therapy targeted at CMV disease and slow, but obvious, clinical response. Her neurological symptoms, both neurocognitive and motor, gradually improved and she began a rehabilitation program. She was transferred to a rehabilitation center and discharged after two months.

Follow up at three months of ART initiation showed CD4 cell count improvement (313 cells/μL (27%)) and undetectable HIV RNA. After 6 months of sustained immune recovery (CD4 cell count 722 cells/μL (24%)) and virologic suppression, maintenance therapy with valganciclovir was stopped.

At 12 months of initial symptoms, she showed no neurocognitive impairment and was able to return to her normal daily activities. Motor strength improved globally to normality besides grade 4/5 in right-hand fingers extension and in the lower limb extension; pin-prick sensibility remained impaired in the right upper limb. She reported neuropathic pain in the lower limbs, which was managed with pregabalin 225 mg twice daily. EMG was consistent with nerve regeneration in the upper limbs and, to a lesser extent, in the right lower limb.

## Discussion and conclusions

In this patient, the evidence of disseminated CMV disease and the presentation with asymmetric neurological signs, with EMG findings consistent with MNM, was sufficient for the clinical diagnosis of CMV MNM.

CMV mononeuritis multiplex can be extensive, involving several limbs or cranial nerves. Both a classic presentation with painful, progressive, multifocal deficits and a more rapidly progressive syndrome, involving multiple nerve distributions, have been described in patients with advanced AIDS [7]. EMG is key in the confirmation of clinical findings, typically showing a neuropathy with multifocal demyelination and axonal loss [2, 7, 12]. While not necessary for a clinical diagnosis of CMV MNM, polymerase chain reaction (PCR) detection of CMV DNA in CSF has been shown to correlate with all forms of CMV neurological disease (sensibility and specificity of > 90%) [13, 14], even in cases of isolated peripheral neuropathy [14]. PCR of plasma or whole blood is also valuable as it can be used as a sensitive “surrogate marker” of CMV reactivation and subsequent disease and is less prone to false negatives compared to antigenemia assays [15, 16]. Moreover, serial quantitative PCR testing of specimens is helpful to monitor response to treatment [15]. Nerve biopsy may provide additional confirmation as CMV has been demonstrated in macrophages, fibroblasts, and endoneurial cells in the superficial nerves of patients with MNM [17]. In our patient, CMV DNA detection in the CSF coupled with signs of altered mental status suggested a more extensive neurologic involvement of CMV disease. However, simultaneous CMV encephalitis and HIV encephalopathy were considered since HIV RNA was also detected in CSF and MRI findings were consistent with the latter.

The diagnosis of CMV encephalitis can be particularly challenging. Patients often present with progressive altered mental status which may be difficult to distinguish from HIV encephalopathy [6, 8, 9]. In contrast to HIV encephalopathy, CMV encephalitis has a more rapid onset (mean onset of less than four weeks) and symptoms of delirium, confusion, apathy, and withdrawal are more frequent [6, 8]. Yet, other non-distinguishing neurological manifestations, such as forgetfulness, memory impairment, and psychomotor slowing, are also common [6]. A more distinct type of CMV encephalitis characterized by ventriculoencephalitis has also been described and presents with rapidly progressive confusion and lethargy [6, 8], with variably associated radiculopathy and cranial nerve deficits [8]. MRI may show multiple hypertense foci distributed widely in the brain or periventricular enhancement on T2-weighted images, although these findings are inconsistently present and largely non-specific [18, 19]. Conversely, HIV encephalopathy typically shows widespread hyperintense lesions on T2-weighted/FLAIR, localized bilaterally in the deep white matter [18], a picture that more closely resembled the MRI findings in our patient. Similarly to CMV neurological disease, HIV RNA detection in CSF correlates significantly (albeit weakly) with the presence of HIV encephalopathy in untreated patients, an association not seen in patients on ART [20, 21]. However, CNS opportunistic infections may also increase intrathecal HIV replication [22].

Treatment of all forms of CMV neurologic disease is similar, although data are scarce. Most authors recommend ganciclovir or foscarnet, or a combination of both drugs. Combination therapy may be considered in those previously treated with CMV-directed drugs (hence with risk of drug-resistant virus) and in patients with disease progression under monotherapy [8]. Foscarnet levels in CSF vary widely, achieving 0 to 3.4 times of plasma concentration (mean values of 23%) [23]. A study of the pharmacokinetics of ganciclovir in plasma and CSF in a nonhuman primate model showed that the drug penetrates into the CSF following IV administration (CSF to plasma area under the curve of 15.5%+/− 7.1%) [24]. Therefore, another rationale for combination therapy use is to increase CSF penetration due to the variable levels achieved by either drug alone. Despite being relatively well tolerated, this regimen may be limited by increased side effects, particularly bone marrow suppression with ganciclovir and nephrotoxicity with foscarnet [23, 25]. There are no data on CSF penetration of cidofovir and therefore it should be avoided [8, 25]. Optimal duration of initial therapy is unknown and should be guided by an improvement of clinical symptoms [6, 8, 9]. Our patient was treated with combination therapy considering the severity of the disease, extensive neurological involvement and slow initial improvement with ganciclovir alone.

Additionally, as with other opportunistic infections, initiation of ART and reversal of profound immunodeficiency is considered essential to successful management [26, 27]. Optimal timing for ART initiation is not well defined and the decision to start ART early in the course of CMV disease must be weighed against the risk of CMV immune reconstitution inflammatory syndrome (IRIS). Nonetheless, data on CMV IRIS presenting as neurological disease are limited: a case of CMV encephalitis was reported in an HIV-infected patient with CMV colitis on ART and valganciclovir (although with adherence issues), where IRIS may have played a role [28]. Reports of immune recovery uveitis following early ART initiation in patients with CMV retinitis are more commonly described throughout the literature [29–31]. Considering the extensive involvement of CMV disease in our patient, ART was initiated after an obvious clinical improvement with combination therapy for CMV was observed.

Optimization of ART using drugs that penetrate effectively into the CSF has been shown to improve the outcome of patients with neurological symptoms and detectable HIV viral loads in the CSF [32, 33]. The possibility of associated HIV encephalopathy motivated our choice to include dolutegravir in the ART regimen since concentrations in the CSF have been shown to be similar to unbound plasma concentrations, achieving therapeutic levels in the CNS [34].

This patient outcome was favorable: clinical improvement was obvious, CMV viremia was cleared and no significant side effects were observed. In the pre-ART era, outcomes of CMV-related neurological disease were generally poor, with a reported median survival of less than three months [6, 9]. Furthermore, CMV MNM was described with simultaneous encephalitis, polyradiculopathy or retinitis leading to death within a few days or weeks if left untreated [6]. The improved survival of HIV-infected patients and decreasing incidence of CMV disease in recent years denotes that prognosis is dependent on adequate antiretroviral treatment and immune reconstitution. The impact of the choice of ART regimen on the patient neurological improvement (besides the obvious benefit of immune recovery) is difficult to ascertain. CSF evaluation at follow-up might have been valuable to determine the effect in CSF HIV viral load since CNS compartmentalization of HIV has also been reported [32].

Duration of maintenance therapy for CMV following neurological disease is debatable. However, considering both the risk of recrudescence of a serious disease and the risk of IRIS, awaiting immune reconstitution (i.e., a sustained rise of CD4 cell count ≥100–150/μL and undetectable HIV RNA for more than 6 months) may be wise before stopping maintenance therapy [26, 27].

In conclusion, CMV neurological disease is increasingly uncommon since the introduction of ART. This patient with AIDS presented predominantly with a clinical picture of MNM, that harbored a disseminated CMV disease. The widespread availability of highly sensitive and specific PCR techniques can significantly shorten the time to diagnosis of CMV reactivation. While the spectrum of neurological involvement of CMV disease in HIV-infected patients can be diverse, it is associated with high morbimortality and risk of rapid progression if left untreated. Since CSF penetration of the available antivirals varies widely, combination therapy with ganciclovir and foscarnet should be considered. The risk of IRIS should be weighed in when deciding the timing of ART initiation and the overlap in manifestations of CMV and HIV infection of the CNS may also be a factor during the selection of ART regimens, favoring drugs with higher CSF penetration efficacy. Nonetheless, ART initiation and subsequent immune recovery were essential for the favorable outcome reported, which contrasted vastly from the natural course of CMV neurological disease in the pre-ART era.

## Abbreviations

AIDS: Acquired immunodeficiency syndrome
ART: Antiretroviral therapy
CMV: Cytomegalovirus
CNS: Central nervous system
CSF: Cerebrospinal fluid
EMG: Electromyography
HIV: Human deficiency virus
IRIS: Immune reconstitution inflammatory syndrome
MNM: Mononeuritis multiplex
PCR: Polymerase chain reaction
